# Our Philadelphia Letter

**Published:** 1885-12

**Authors:** Harry Huzza

**Affiliations:** Philadelphia


### OUR PHILADELPHIA LETTER.

THE MEDICAL COLLEGES—GEORGIA STUDENTS IN ATTENDANCE—THE CLINICS—WRITTEN EXAMINATIONS HEREAFTER IN JEFFERSON—THE PURITY OF PHILADELPHIA WATER QUESTIONED—FAIRMOUNT PARK AND ITS BEAUTIES—THE JOURNAL, ETC.

*Editors Atlanta Medical and Surgical Journal:*

I have nothing of importance to chronicle this month. There seems to be a lull in matters, medical and otherwise, so that everything is moving very quietly. Even the Medical Congress has dropped out of notice and discussion. The students are all in and are busy in the winter study. Georgia is represented by seven students in the colleges here. It is probable that three of these will graduate in the spring. The clinics have been rather dull. Though still instructive, yet they present no special attraction. Minor operations seem to rule the day.

In my first letter I mentioned two cases of aphasia, diagnosed as resulting from injury to the left third frontal convolution of the brain. A case in a late clinic was of interest in this connection. The patient, a boy of ten, was struck two years ago over the left eye about an inch and a half back of the upper and outer orbital margin with a pitchfork, which penetrated the skull. Was unconscious nearly a week. Vomited first day, but not afterwards. Lost completely the power of speech and presented right-sided hemiplegia. Gradually he regained command of words; the paralysis also became less marked; but he has not yet obtained complete control of his muscles, nor is his speech perfect. The blow in this case was anterior and superior to the junction of the spheno-parietal and coronary sutures, just over the speech center of Broca. This is the position of the third anterior convolution. So here was an accidental experiment, showing that an injury in that place would, as previously diagnosed and demonstrated, produce aphasia and right hemiplegia.

There was also exhibited an illustrative case of *spina bifida* in a baby of two years. Tumor was as large as a peck measure. It seems to be the present teaching to let such cases alone, as operative interference is almost invariably fatal. The specimens of a curious case were also seen. An engineer, running to catch his train, whirled around a post by resting one hand on it. A ring on his third finger caught in a nail, and as he passed on rapidly, he left behind the finger, pulling out the long flexor tendon and its entire muscular attachment. So quickly did it occur that he felt no pain and did not know it till the blood was noticed.

The operation for rapid dilatation of the cervix in dysmenorrhœa continues to grow in favor. Its results have been most satisfactory. I saw a very typical, yet a difficult case that may be of interest. The patient, a girl with persistent dysmenorrhœa, menses at present delayed, had eruption of bullæ on face, was very anæmic, cervix uteri small, conical, and "pin-hole" in size. While pointing it out as a suitable case for rapid dilatation, the doctor remarked that "unless a man dared to dilate over an inch, and dared skillfully, he would certainly fail of success;" that he had performed the operation over two hundred times, and had as yet seen no ill results follow. Though the cervix in this case was so small that a special dilator had to be first used before the ordinary size could be introduced, he dilated to an inch and a quarter in less than eight minutes.

As an item of perhaps medical interest, at least of hygienic importance, there has been some agitation about the purity of the city water. It is probable that nothing will be done, however, till next summer's heat shall arouse attention. However, it is needed, for I noticed several sewers emptying into the Schuylkill just above the pumping stations. But whatever the dangers in a sanitary point of view when large masses of people are pent up within the confines of a great city, Philadelphia has solved one side of the question most satisfactorily. The utility of fresh air and contact with nature cannot be denied. In Fairmount Park is provided such a luxury for the population. Extending nine miles up the river, with every diversity of hill and dale, yet in easy reach by railroad or street cars, it affords to thousands each week a means of health, amusement and relaxation. Above all, it is a park intended for use, and no staring sign-boards, "keep off the grass," no policemen to guard the trees, nor any other obstructions to one's free enjoyment and recreation are found. Fairmount Park must be seen to be appreciated. But it surely is a benefit from a hygienic standpoint. I only wish our Georgia cities would follow the example.

To return to the colleges, Jefferson has instituted several changes this year of which perhaps you may not have heard. The examinations will hereafter be written instead of oral. Theses will not be required except in competition for prizes. Then, even though the regular post-gradute course here has been discontinued, I notice quite a number of practitioners attending the lectures and clinics. Oral examinations and the compulsory thesis were both old landmarks of medical education.

THE JOURNAL is always a welcome visitor, bringing, as it does, news from Georgia. With many wishes for its continued prosperity,

Truly yours,

HARRY HUZZA.

Philadelphia, November 20, 1885.

